# Epigenetic Regulation in Ischemic Neuroprotection: The Dual Role of HDACs and HATs in Neuroinflammation and Recovery

**DOI:** 10.3390/antiox14081015

**Published:** 2025-08-19

**Authors:** Malwina Lisek, Natalia Bochenska, Julia Tomczak, Julia Duraj, Tomasz Boczek

**Affiliations:** Department of Molecular Neurochemistry, Medical University of Lodz, 92-215 Lodz, Poland; natalia.bochenska@umed.lodz.pl (N.B.); julia.tomczak1@student.umed.lodz.pl (J.T.); julia.duraj@student.umed.lodz.pl (J.D.); tomasz.boczek@umed.lodz.pl (T.B.)

**Keywords:** histone deacetylase, neuroinflammation, ischemia

## Abstract

Ischemic brain and retinal injuries trigger complex molecular cascades involving neuroinflammation, oxidative stress, and neuronal death. Among these mechanisms, epigenetic regulation has emerged as a critical modulator of the injury response. Histone deacetylases (HDACs) and histone acetyltransferases (HATs) dynamically control gene expression by altering chromatin structure. HDACs often promote neuroinflammation and neuronal apoptosis through repression of neuroprotective and anti-inflammatory genes, while HATs generally enhance the transcription of genes involved in cell survival and repair. In ischemia, specific HDAC isoforms (e.g., HDAC1, HDAC2, HDAC3, and HDAC6) have been implicated in microglial activation, glial reactivity, and disruption of immune balance. Conversely, HATs such as CBP/p300 and Tip60 contribute to neuronal resilience and immune regulation. Understanding the dual and context-dependent roles of these epigenetic enzymes offers promising therapeutic avenues. Selective HDAC inhibitors or HAT activators may represent novel strategies to mitigate ischemic damage, support neuroprotection, and facilitate functional recovery.

## 1. Introduction

The fragile balance in our bodies may be easily disrupted by sudden events such as a blockage in blood vessels causing local restriction in blood supply, hypoxia, cell malnutrition, and cytotoxicity—a condition known as ischemia [1]. Ischemia can affect any part of the body, most commonly the heart, brain, bowels, limbs, or kidneys. When blood flow to the entire brain is compromised, typically by cardiac arrest, severe hypotension, or asphyxia, the condition is referred to as global brain ischemia. In contrast, focal ischemia occurs when a reduction in blood supply is localized to a specific region of the brain due to the blockage of a single cerebral artery, most commonly caused by ischemic stroke [2]. This review focuses on brain and retinal ischemia and aims to summarize current breakthroughs that have significantly contributed to our understanding of the epigenetic pathways in ischemic pathology.

Ischemic stroke, also known as cerebral infarction, an acute form of cerebral ischemia, is a sudden occlusion of blood flow due to a thrombus or embolus [3]. Acute ischemia can also occur as a transient ischemic attack (TIA), where symptoms resolve within 24 h [3]. The opposite condition, chronic cerebral ischemia, may be caused by long-term disease, e.g., atherosclerosis [4]. Ischemic events affecting cerebral circulation can simultaneously or secondarily impair retinal circulation. The retina requires a constant and rich blood supply from the central retinal artery and choroidal circulation; the deficiency of oxygen and nutrients may result in damage to retinal neurons, especially retinal ganglion cells (RGCs), leading to vision impairment or even blindness [5]. Retinal ischemia may occur as an acute condition due to central retinal artery occlusion or a chronic condition as in diabetic retinopathy and retinal vein thrombosis [5].

Ischemia pathology involves multiple interconnected mechanisms, primarily neuroinflammation, oxidative stress, and excitotoxicity [6]. These processes depend on the onset, progression, and severity of ischemic injury, particularly for vulnerable cells like hippocampal and retinal neurons [6]. Neuroinflammation triggered by ischemia activates central nervous system (CNS) immune residents—microglia and astrocytes—and promotes the infiltration of peripheral immune cells, like monocytes/macrophages, neutrophils, and T cells. This is followed by the production of pro-inflammatory cytokines and the upregulation of adhesion molecules on endothelial cells in the affected area [7]. Neuroinflammation amplifies cellular damage; however, this response is also an initial signal of recovery, repairing the brain by stimulating neuroplasticity or the recovery of motor dysfunctions [8]. The oxidative burst, enhanced by neuroinflammation, disrupts the balance between the production of reactive oxygen species (ROS) and the capacity of antioxidant defenses, causing cell stress [8]. Rapid ROS generation, especially by the activation of NADPH oxidase (nicotinamide adenine dinucleotide phosphate oxidase), xanthine oxidase, immune cell activity, and mitochondrial malfunction, leads to membrane lipid peroxidation, DNA fragmentation, and protein oxidation, and it can even activate apoptotic and necrotic pathways [9]. ROS production and neuroinflammation can be further amplified by excitotoxicity, which arises from excessive activation of glutamate receptors. Ischemia-induced depletion of adenosine triphosphate (ATP) impairs glutamate reuptake, leading to extracellular accumulation of glutamate. This overactivates NMDA (N-methyl-D-aspartate) and AMPA (α-amino-3-hydroxy-5-methyl-4-isoxazolepropionic acid) receptors, causing massive Ca^2+^ influx [10]. Due to the high density of ionotropic glutamate receptors and overall high metabolic activity, hippocampal neurons and RGCs are especially vulnerable to excitotoxic damage [11,12].

The cellular response to ischemic damage depends largely on epigenetic mechanisms, which are severely affected by ischemia. Histone acetylation, regulated by histone acetylases (HATs) and histone deacetylases (HDACs), is decreased in ischemia; therefore, the transcription of protective genes is repressed due to limited accessibility in condensed chromatin [13]. The dynamic balance between HATs and HDACs represents a fundamental regulatory mechanism of gene expression in the brain, particularly under conditions of ischemic stress. While HDACs are generally associated with chromatin condensation and transcriptional repression, HATs promote a relaxed chromatin structure through acetylation of histone tails, facilitating the transcription of genes involved in cell survival, repair, and stress adaptation. Disruption of this epigenetic balance contributes to neuronal vulnerability, whereas restoring acetylation homeostasis has been shown to promote neuroprotection [14,15]. HATs are distinguished depending on their cellular localization: type A HATs are nuclear and acetylate histones within chromatin and other chromatin-associated proteins, and type B HATs are cytoplasmic HATs, which have a direct impact on transcription, acetylating newly synthesized histones. Type A HATs can be further classified into five families based on their sequence similarity: nuclear receptor coactivators and general transcription factors, p300/CBP (CREB (cAMP-response element binding protein)-binding protein), GNAT (GCN5 (general control of nuclear-5)-related N-acetyl transferases), MYST (MOZ (monocytic leukemia zinc-finger protein), YBF2 (yeast binding factor 2)/SAS3 (something about silencing 3) and SAS2, and TIP60 (Tat interactive protein-60) [16].

HDACs (classes I–IV) are widely expressed in the central nervous system (CNS), with isoform-specific distributions across different brain regions and cell types [17]. All eleven classical HDAC isoforms (HDAC1–11) have been detected in the mouse cortex, highlighting their broad involvement in CNS function. Their expression is dynamically regulated in response to physiological and pathological stimuli, including ischemia, which alters HDAC levels in a region- and cell-specific manner. HDAC activity is dynamically regulated during ischemia, reflecting the complex and time-dependent epigenetic response of the brain to hypoxic–ischemic stress. Ischemia triggers rapid and region-specific alterations in HDAC expression, localization and enzymatic function, which in turn modulate key cellular processes such as apoptosis, inflammation, oxidative stress, and metabolic adaptation [18]. Other epigenetic regulators, such as histone methyl transferase (HMT), are also upregulated in stroke models [19,20], and increased methylation correlates with reduced levels of neuroprotective genes [21,22]. Histone modifications, through controlling gene expression, may shift the balance towards injury or recovery.

DNA methylation generally decreases gene transcription through a chemically stable modification catalyzed by DNA methyltransferase (DNMT). The addition of a methyl group (-CH_3_) at the 5′ position of cytosine within a gene promoter prevents the binding of transcription factors to their specific binding sites [23]. Ischemia induces global DNA hypermethylation, silencing critical genes involved in neuronal survival and anti-inflammatory responses [23,24]. Moreover, DNMT1 and DNMT3a are overexpressed in ischemic brain regions [25,26]. Recent studies have shown that DNMT inhibitors (such as, 5-aza-2′-deoxycytidine) can restore gene expression and improve outcomes in experimental models [25,27].

RNA-based mechanisms, including microRNAs (miRNA), long non-coding RNAs (lncRNA), and circular RNAs (circRNA), are disrupted at different stages of stroke, suggesting their potential as prognostic or therapeutic tools [22]. MicroRNAs—small single-stranded RNAs (18 to 22 nucleotides)—silence target genes by promoting mRNA degradation or by inhibiting translation [22]. Several miRNAs have been identified as dysregulated in ischemia, including miR-124, miR-21, miR-210, miR-211, and miR-335 [22].

Many of these are considered potential circulating biomarkers of stroke. Long non-coding RNAs (transcripts longer than 200 nucleotides) modulate chromatin structure and gene expression [28]. Over 200 lncRNAs have been found to be deregulated in animal models of ischemia and in the blood of ischemic patients [29]. For instance, MALAT1 (metastasis associated lung adenocarcinoma transcript 1) is upregulated in ischemic models and affects endothelial cell survival and inflammation [29]. Emerging regulators of miRNA activity and gene expression, such as circular RNAs, remain under investigation in the context of ischemia [30].

Notably, because epigenetic modifications are reversible, they offer promising opportunities for the development of novel therapeutic approaches for ischemia in all its stages.

## 2. HDACs and Neuroinflammation in Ischemia

Cerebral and retinal ischemia progress through a series of pathological phases [31]. The acute stage of ischemia features neuronal necrosis, followed by calcium influx, excitotoxicity, and oxidative stress during the subacute phase [32]. In the delayed injury phase, inflammatory responses are initiated, along with neuronal regeneration [33]. Neural tissue is divided into the “ischemic core”, where neurons die due to rapid ATP depletion and associated processes including Na+/K+-ATPase failure and Ca^2+^ overload [34,35], and the “ischemic penumbra,” where neurons remain alive [35]. Every stage of the ischemic response is affected by post-ischemic neuroinflammation [35]. In ischemia, microglia, gliosis, and neuroinflammation are closely linked and play critical roles in determining the degree of neuronal damage and recovery. In the infarct core, the number of microglia declines, whereas in the penumbra, their numbers peak 48–72 h post-stroke, showing a correlation between microglia dynamics and neuronal injury [36,37].

Microglia are the central nervous system’s resident innate immune cells, acting as sentinels that monitor for any pathological changes in the brain environment [32]. In response to ischemia, early activation occurs within the range of minutes to hours, leading to morphological changes and migration toward the injury [38]. During activation microglia undergo dynamic morphological transformation, increasing soma size and shortening cytoplasmic processes [39]. When activated, microglia can adopt two distinct phenotypes—pro-inflammatory and neurotoxic (M1-like) phenotypes—secreting cytokines tumor necrosis factor-alpha (TNF-α), interleukin-6 (IL-6), and interleukin-1β (IL-1β), ROS, and nitric oxide (NO), triggered by the toll-like receptor (TLR) and interferon-γ (IFN-γ) signaling pathways [40]. In later phases or under therapeutic modulation, they may shift to an anti-inflammatory (M2-like) phenotype, releasing cytokines interleukin-4 (IL-4) and interleukin-10 (IL-10), nerve growth factor (NGF), insulin-like growth factor-1 (IGF-1), and neurotrophic factors including glial cell-derived neurotrophic factor (GDNF) and brain-derived neurotrophic factor (BDNF) [40]. Dysregulation (e.g., prolonged M1 state) leads to chronic inflammation and worsens injury. Microglia activation is a double-edged sword—initially protective by clearing debris, but exacerbating injury upon prolonged activation [32]. While direct mechanistic links in ischemia models are emerging, HDAC-driven microglial activation is known to upregulate cytokines such as TNF α, IL 1β, IL 6, and chemokines C-C motif ligand 2 (CCL2) and C-X-C motif chemokine ligand 10 (CXCL10), which are common hallmarks of neuroinflammatory states [41,42]. In fact, immediate suberoylanilide hydroxamic acid (SAHA) treatment, a pan-HDAC inhibitor, after transient focal cerebral ischemia (tMCAO) resulted in reduced infarction volume and improved outcomes [41]. This improvement was accompanied by the suppression of M1 cytokine expression, such as IL-6, TNF-α, and inducible nitric oxide synthase (iNOS), while supporting the transcription of M2 cytokines (Arg-1 and IL-10) in lipopolysaccharide (LPS)-challenged mouse microglia, an shifting microglia from pro-inflammatory CD86 (M1 markers) to anti-inflammatory CD206 (M2 marker) in the early stage of post-stroke neuroinflammation [41].

HDAC1 has also been implicated in microglial inflammatory responses in ischemia, contributing to neuroinflammation and blood–brain barrier (BBB) breakdown. Knockdown or inhibition of HDAC1/2 in microglia suppresses LPS-stimulated expression of pro-inflammatory cytokines IL-6 and TNF-α. In addition, loss of HDAC1 leads to compensatory upregulation of HDAC2, indicating overlapping roles in regulating inflammation. HDAC1 is upregulated early in ischemic penumbra but shows mixed localization, indicating that its role in apoptosis seems to be less decisive than that of HDAC2 [43]. Additionally, results obtained in a rat stroke model showed that HDAC1 dysfunction could promote BBB damage through the destruction of tight junction proteins, such as ZO-1 (Zonula occludens-1) and occludin. HDAC1 inhibition also **increases** levels of astrocyte and microglial gliosis, TNF-α, IL-1 beta, lactate dehydrogenase, and ROS, then triggering metalloproteinases MMP-2 and MMP-9 activity [43].

However, although studies on the role of HATs are still limited, some evidence suggests their potential as anti-neuroinflammatory factors. Acetyltransferase activity can modify neuroinflammatory signaling pathways, CBP/p300 acetylates p65 subunit of NF-κB, modulating transcriptional activity at neuroinflammatory gene promoters (e.g., IL 6, cyclooxygenase (COX-2), iNOS), balancing pro- and anti-inflammatory responses [44]. In a microglial autophagy context, CBP/p300-mediated acetylation regulates the NF-κB/NLRP3 (nucleotide-binding oligomerization domain (NOD)-like receptor pyrin domain containing 3) pathway, linking histone modifications to inflammasome activity [44]. During neuronal apoptosis (e.g., in ischemic injury models), CBP/p300 is cleaved by caspases-6, leading to decreased histone acetylation and impaired transcription of neuroprotective genes. This reduction in CBP/p300 contributes to deregulated neuroinflammatory responses and worsened neuronal survival in ischemic or degenerative contexts [45]. Gliosis refers to reactive changes in glial cells—primarily astrocytes—and in the retina—Müller cells. Ischemia-induced damage and signals from dying neurons trigger astrogliosis, which manifests as upregulation of GFAP (glial fibrillary acidic protein) and secretion of pro-inflammatory cytokines, contributing to excitotoxicity by impairing glutamate uptake and release. In chronic phases, astrocytes form a glial scar, which isolates damaged areas but also inhibits axonal regeneration. Among histone deacetylases, HDAC2 has been shown to participate in astrocyte reactivity. Post-ischemic increases in HDAC2 in astrocytes, after subarachnoid hemorrhage, coincide with downregulation of GLT-1 (glutamate transporter), promoting excitotoxicity. Interestingly, HDAC2 inhibition with SAHA or MS-275 (class I inhibitor) restores GLT-1 levels, enhancing glutamate clearance and reducing excitotoxic injury in white matter and retinal models [46,47,48]. HDAC2 levels increase in neurons and astrocytes 4–24 h after stroke, driving deacetylation of histone protein H3 at the ninth lysine residue (H3K9) and contributing to apoptosis and astrocyte reactivity, supported by co-localization of HDAC2 with TUNEL (Terminal deoxynucleotidyl transferase dUTP Nick End Labeling)-labeled cells in the penumbra photothrombotic stroke (PTS) being induced [49].

### Retinal Neuroinflammation

**Figure 1:**
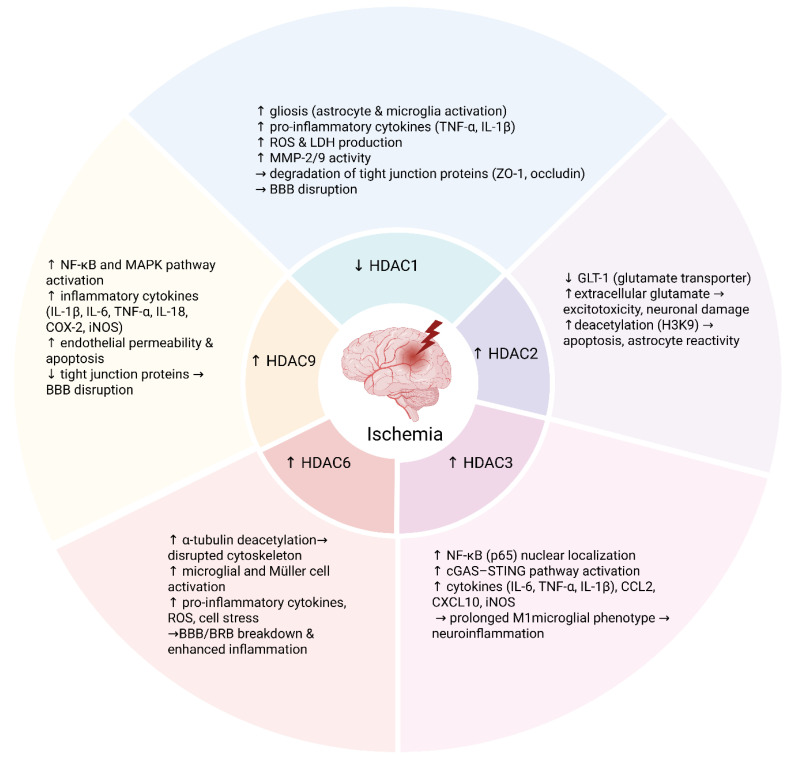
Schematic summary of the roles of HDAC isoforms in neuroinflammation and blood–brain/retinal barrier dysfunction following ischemia. Distinct HDACs modulate post-ischemic neuroinflammatory responses and blood–brain barrier (BBB) or blood–retinal barrier (BRB) integrity through isoform-specific mechanisms. Decreased HDAC1 activity promotes gliosis (activation of astrocytes and microglia) and increases production of pro-inflammatory cytokines such as TNF-α and IL-1β, ROS, and MMP-2/9, resulting in tight junction degradation and BBB disruption. HDAC2, upregulated in astrocytes after ischemia, enhances glutamate-mediated excitotoxicity and astrocyte reactivity via deacetylation of histone H3K9. HDAC3 promotes microglial pro-inflammatory activation by facilitating NF-κB and activating the cGAS–STING pathway, sustaining a pro-inflammatory (M1) phenotype. HDAC6 destabilizes the cytoskeleton through deacetylation of α-tubulin, thereby amplifying glial activation, oxidative stress, and barrier dysfunction. HDAC9 activates the NF-κB and MAPK signaling pathways, increasing the expression of inflammatory mediators, endothelial permeability, and tight junction protein loss. Collectively, these isoform-specific mechanisms highlight the epigenetic regulation of inflammation and vascular injury after ischemic insult. Created in BioRender. Tomczak, J. (2025) https://BioRender.com/2636j09 (accessed on 3 August 2025).

The retina is composed of multiple layers—the ganglion cell layer (GCL), located more internally, and the axons of these RGCs form the optic nerve, which transmits visual information to the brain. Simultaneously, Müller cells overlay these layers, which are responsible for structural support, ion balance, and interaction with microglia in the retina [50]. Retina-specific Müller gliosis in response to ischemia is characterized by upregulation of GFAP and cellular hypertrophy, releasing vascular endothelial growth factor (VEGF), which worsens edema and neovascularization [50]. Recent studies have shown that increased retinal HDAC activity, followed by protein hypoacetylation, contributes to the injury response induced by ischemia [51]. Multiple preclinical studies in retinal models strongly support the broad involvement of class I and II HDACs in Müller cell activity and gliosis after injury. After retinal ischemia–reperfusion (RIR), total class I HDAC activity—specifically HDAC1/2—increases significantly, leading to histone hypoacetylation, neuronal damage, and Müller cell dysfunction. Ischemic preconditioning (IPC) prevents this rise in HDAC1/2, supporting Müller cell and retinal neuron survival, partly by suppressing HDAC activity [51]. HDAC3 and HDAC6 are constitutively expressed in the retina and are implicated in Müller cell-mediated gliosis and neuroinflammation. Inhibition of HDAC3 with RGFP966 protects against diabetic retinopathy, reducing Müller cell activation as indicated by GFAP upregulation [52]. Liu et al., in a rat model of RIR injury, demonstrated that HDAC6 expression increases significantly following injury. Inhibition of HDAC6 using the selective inhibitor tubacin preserved retinal morphology and RGC survival, reduced pro-apoptotic and inflammatory markers, and supported autophagy and antioxidant pathways. Interestingly, the neuroprotective effect was associated with increased acetylation of α-tubulin rather than histone acetylation, highlighting HDAC6′s cytoplasmic regulatory role [53]. In a rat RIR model, trichostatin A (TSA) suppressed the increase in retinal TNFα and blocked the secretion of MMP 1/3, expressed by Müller glia and astrocytes. Immunochemistry results confirmed that TSA restored histone H3 acetylation and attenuated glial inflammatory signaling. The same study showed that TSA treatment significantly improved electrophysiological parameters seven days after ischemia, preserving the inner retinal layers and structural integrity. These functional improvements were confirmed in both rat and mouse retinal ischemia models, including in Hdac2^+^/^−^ mice, underscoring TSA’s protective effect against histone hypoacetylation and retinal degeneration [54]. A graphical summary of HDACs’ role in neuroinflammation can be found in Figure 1.

## 3. HDACs and Macrophage Activation in Ischemia

Ischemia triggers neuronal death together with glial activation, followed by cytokine release and the attraction of peripheral immune cells. These cells, including neutrophils, monocytes, and lymphocytes, are recruited from the bloodstream to the injury site via the compromised blood–retinal barrier (BRB) or blood–brain barrier (BBB), contributing to the neuroinflammatory response and further tissue damage. The earliest responders, neutrophils, secrete proteolytic enzymes and reactive oxygen species, driving oxidative stress. Monocytes transform into macrophages upon migration to the injury site, releasing pro-inflammatory cytokines and phagocytosing cellular debris. Lymphocytes, predominantly T cells, also play a substantial role in moderating the immune response [50].

HDAC3 plays a pivotal role in regulating macrophage polarization and neutrophil infiltration, thereby influencing neuroinflammatory processes during ischemic injury. HDAC3 functions as an “epigenomic brake” on alternative (M2) macrophage activation. In vitro studies on bone marrow-derived macrophages show that HDAC3 deletion leads to increased expression of M2 markers (e.g., arginase 1, chitinase 3-like-3, dectin-1) and decreased expression of pro-inflammatory (M1) markers (e.g., IL-6, macrophage inflammatory protein-1 alpha) [55]. HDAC3 expression increases selectively in microglia following tMCAO, driving pro-inflammatory signaling via innate immune cyclic guanosine monophosphate–adenosine monophosphate (sGAS)—a stimulator of interferon gene (cGAS-STING) pathways and deacetylation―causing nuclear localization of NF-κB p65 (nuclear factor kappa-light-chain-enhancer of activated B cells) [42,56]. Microglia-specific deletion or pharmacological inhibition of HDAC3 reduces expression of TNF α, IL 1β, IL 6, and iNOS, represses NF-κB targets, shifts the microglial phenotype toward an anti-inflammatory state, and improves stroke outcomes [41]. Microglia isolated after tMCAO show strong upregulation of IL 6, CCL2, and CXCL10—all reduced upon HDAC3 inhibition [57]. Additionally, in a mouse model of middle cerebral artery occlusion–reperfusion (MCAO/R) and a model of microglial oxygen–glucose deprivation/reperfusion (OGD/R), the administration of pterostilbene (PTS), a pan-HDAC inhibitor, led to a decrease in HDAC3 expression and activity, increased acetylation of nuclear factor (erythroid-derived 2)-like 1 (Nrf1) in the nucleus, and repressed the interaction of Nrf1 with p65 and p65 accumulation. This resulted in reduced infarct size and neuroinflammation (iNOS/Arg1, TNF-α, and IL-1β levels) after ischemic stroke [58].

New genomic analyses have revealed that HDAC3 directly represses M2 genes via deacetylating histone tails like H3K9 and H3K27, maintaining macrophages in a pro-inflammatory state [55]. Recent studies have reported that myeloid cells upregulate HDAC3 in a mouse model of RIR injury [59]. In myeloid-specific HDAC3 knockout (M-HDAC3 KO) and floxed control mice subjected to retinal IR, retinas of M-HDAC3 KO mice showed lower proliferation and infiltration of myeloid cells. Interestingly, myeloid cells lacking HDAC3 more effectively engulfed apoptotic cells in vitro and after RIR injury in vivo compared to wild-type myeloid cells, suggesting that HDAC3 delays the reparative phagocytosis of dead cells—known as efferocytosis. This is thought to be partially associated with the upregulation of the reparative enzyme arginase 1 (A1) in HDAC3 KO macrophages, which enhances efferocytosis. In addition, treatment of wild-type mice with the HDAC3 inhibitor RGFP966 reduced the retinal neurodegeneration and thinning caused by IR injury [60]. HDAC3 deficiency or inhibition enhances macrophage responsiveness to IL-4, promoting a shift toward anti-inflammatory M2 phenotypes, even in the presence of pro-inflammatory signals—indicating dual regulation through M1 support and M2 suppression [55]. Furthermore, pharmacological HDAC3 inhibitors in monocyte/macrophage models dampen LPS-induced cytokine production of IL-1β, indicating reduced innate inflammatory signaling [61].

**Figure 2:**
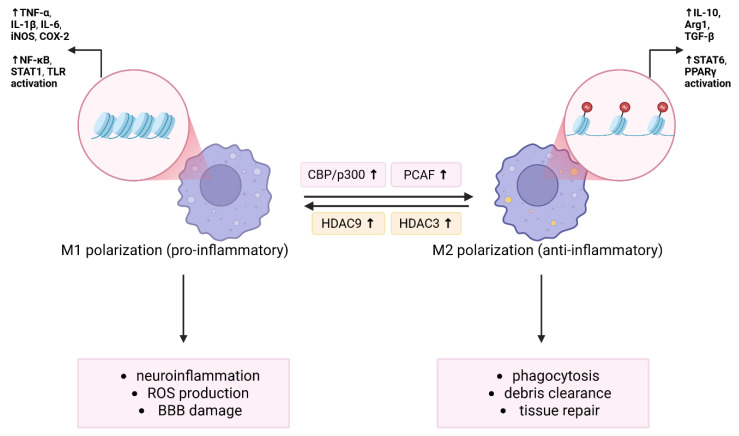
Epigenetic regulation of microglia/macrophage polarization via HDAC and HAT activity. Histone acetylation and deacetylation dynamically regulate the polarization of microglia and macrophages in response to ischemic injury. Pro-inflammatory M1 polarization is promoted by increased HDAC activity, particularly HDAC3 and HDAC9, which enhances the expression of inflammatory genes such asTNF-α, IL-1β, IL-6, inducible nitric oxide synthase (iNOS), and COX-2 via NF-κB, signal transducer and activator of transcription 1 (STAT1), and Toll-like receptor (TLR) signaling. This promotes neuroinflammation, ROS production, and BBB disruption. In contrast, HATs facilitate anti-inflammatory M2 polarization by promoting acetylation of histones, thereby enhancing the expression of arginase 1 (Arg1), IL-10, and transforming growth factor beta (TGF-β) through signal transducer and activator of transcription 6 (STAT6) and peroxisome proliferator-activated receptor gamma (PPARγ) activation. M2 macrophages/microglia support phagocytosis, debris clearance, and tissue repair. The balance between HDAC and HAT activity shapes the immune microenvironment and outcomes after ischemia. Created in BioRender. Tomczak, J. (2025) https://BioRender.com/o3x5sa5 (accessed on 3 August 2025).

Genome-wide association studies have identified a variant in HDAC9 related to large-vessel ischemic stroke, which is linked to enhanced stroke risk by promoting carotid atherosclerosis [62,63]. These results, together with upregulation of HDAC9 expression in the ischemic cerebral hemisphere in rodent models of cerebral ischemia–reperfusion, shed light on this class II HDAC member [64]. The same authors showed that gene silencing with HDAC9 recombinant lentivirus in vivo mitigated cerebral injury in experimental stroke and improved brain microvessel endothelial cell dysfunction caused by increased neuroinflammatory responses, cellular apoptosis, and endothelial cell permeability associated with reduced expression of tight-junction proteins in the oxygen–glucose deprivation model [64]. Other studies also support the role of HDAC9 in ischemic injury response. HDAC9 deletion or silencing leads to smaller infarct volumes, improved neurological outcomes, and suppression of systemic and neuroinflammatory cytokines including IL-1β, IL-6, TNF-α, IL-18, COX-2, and iNOS—mediated in part by reduced activation of NF-κB and the mitogen-activated protein kinase (MAPK) pathway [65]. HDAC9 knockout in low-density lipoprotein (LDL) receptor-deficient mice leads to a shift toward anti-inflammatory (M2) macrophage polarization, lowers expression of neuroinflammatory genes (IL-1β, MCP-1/CCL2), and decreases in atherosclerotic plaque formation [66,67]. Recent findings have shown that upregulation of HDAC9 expression in myeloid cells with deletion of the conserved non-coding CRE 3′ promotes atherosclerosis via HDAC9 binding and deacetylation of NLRP3, which activates the inflammasome and induces pyroptotic cell death. Genetic and pharmacological inhibition of HDAC9 in myeloid cells stabilizes atherosclerotic plaques by reducing necrotic core expansion [68]. In macrophages, HDAC9 epigenetically upregulates toll-like receptor 4 (TLR4) signaling, reinforcing M1 polarization and inflammatory gene expression [69]. Altogether, these results highlight HDAC9′s role in promoting inflammatory macrophage phenotypes and lesion development. Figure 2 presents epigenetic regulation of microglia/macrophage polarization via HDAC and HAT activity.

## 4. HDACs and Adaptive Immunity in Ischemia

Ischemic injury not only triggers innate immune responses but also involves adaptive immunity, particularly T cell subsets. HDACs are key epigenetic regulators that influence T cell function, the balance between pro-inflammatory and regulatory phenotypes, and overall neuroinflammatory outcomes. CD4+ T cells are divided into two subgroups based on their functions: T helper cells (Th) and T regulatory cells (Tregs). Tregs, distinguished by the expression of the fork-head box P3 (Foxp3) transcription factor, moderate the inflammatory environment and maintain immune homeostasis. Tregs can migrate to the injury site and alleviate inflammation by increasing the levels of anti-inflammatory factors and activating macrophages to clear debris [50].

Treatment with TSA in mouse stroke models increased both the number and suppressive function of FoxP3^+^ Tregs. TSA-treated mice showed reduced infarct volumes, improved neurological performance, and decreased pro-inflammatory cytokine expression—effects that depended on Tregs and were mediated by IL-10 [70]. HDAC inhibitors, such as TSA and SAHA, skew CD4^+^ T cells away from pro-inflammatory Th17 differentiation and toward a regulatory phenotype. This shift involves downregulation of IL-6 receptor expression, suppression of signal transducer and activator of transcription 3 (STAT3) phosphorylation, and reduced nuclear receptor retinoid-related orphan receptor gamma T (RORγT) activity [71]. Consequently, T cell-driven inflammation (including Th17-mediated effects) is tempered, contributing to improved outcomes in ischemic injury models. Selective inhibition of HDAC6 impairs the effector functions of CD8^+^ T cells—including activation and cytokine production—as demonstrated in murine skin-inflammation and graft-versus-host disease models [72]. HDAC6 is crucial for proper T cell receptor (TCR) signaling via heat shock protein 90–lymphocyte-specific protein tyrosine kinase (HSP90-LCK) interactions [73]. While these studies are not ischemia-specific, they highlight the critical role of HDACs in controlling CD8^+^ T cell activation and potential inflammatory responses. Data from other tissues suggest that HDAC6 supports CD8^+^ T cell activation and cytokine production. Thus, HDAC6 inhibition could attenuate CD8^+^-mediated tissue damage during post-ischemic inflammation [74]. Although direct studies in ischemia are lacking, findings from HDAC-inhibitor-driven regulation of other T cell subsets (Tregs and Th17) imply that HDAC6 may similarly govern CD8^+^-mediated immune responses. For more information about HDACs and T cell regulation, see this review [75].

After ischemic stroke or RIR, BBB and BRB breakdown permits B cell infiltration into the CNS. HDAC6 broadly regulates B cell activation and B-T cell interactions through modulation of cytoskeletal dynamics and NF-κB acetylation. HDAC inhibitors such as panobinostat and vorinostat repress autoreactive B cell proliferation, reduce autoantibody levels, and mitigate tissue pathology in autoimmune disease models—without significantly impairing B cell memory. These compounds also upregulate miRNAs in B cells that control activation-induced cytidine deaminase (AID) and B lymphocyte-induced maturation protein (Blimp-1) expression, affecting class-switch recombination and antibody production [76,77].

HAT–PCAF and Tip60 also demonstrate roles in T cell differentiation and macrophage function. It was found that PCAF levels rise during M1 macrophage polarization, and that overexpression of PCAF significantly suppresses pro-inflammatory genes (TNF-α, IL-6 and, CXCL10), while PCAF deficiency enhances them [78]. Mechanistically, PCAF dampens the inflammatory response by inhibiting NF-κB signaling and promoting expression of anti-inflammatory Krüppel-like family of transcription factors (KLF2 and KLF4) [79].

Studies on hypoxia/reoxygenation injury have revealed that Tip60 cooperates with myocardin-related transcription factor A (MRTF-A) and H3K4 methyltransferase to drive iNOS transcription in macrophages. This illustrates Tip60′s role in activating pro-inflammatory genes under ischemic stress. Additionally, Tip60 regulates hypoxia-inducible factor 1α (HIF-1α) acetylation under LPS stimulation, contributing to its stabilization and subsequent macrophage inflammatory response [80]. In CD4^+^ T cells, PCAF is essential for repression of transcription factor forkhead box P3 (FOXP3), a master regulator of regulatory T cells, thereby influencing Treg development and immune tolerance [81]. While not ischemia-specific, it seems relevant that general roles of Tip60 include acting as a cofactor for FoxP3, acetylating histones such as H4K16 at FoxP3 target promoters, and stabilizes FoxP3, thus promoting Treg suppressive function and immune homeostasis [82].

## 5. HDACs and Nrf2 Signaling in Ischemia

**Figure 3:**
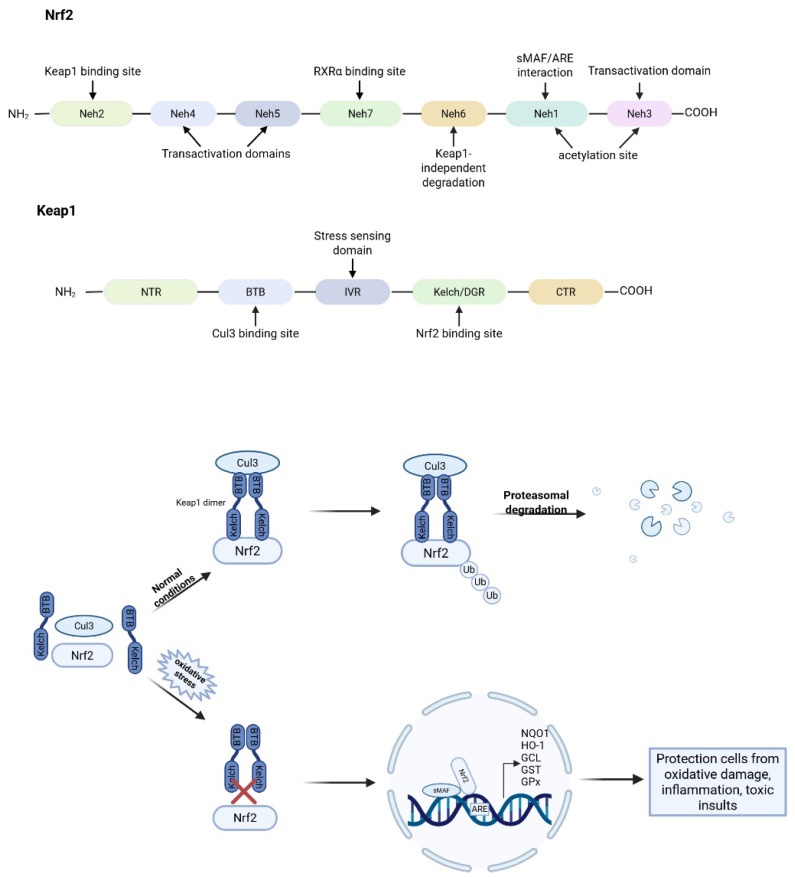
Domain structures of Nrf2 and Keap1, and regulatory mechanism of Keap1 on Nrf2. Under normal homeostatic conditions, Keap1 acts as an adaptor protein linking Nrf2 to the Cul3-based E3 ubiquitin ligase complex, facilitating the rapid ubiquitination and subsequent proteasomal degradation of Nrf2 in the cytoplasm. Under stress (electrophiles, ROS, or endoplasmic reticulum stress) conditions, the interaction between Keap1 and Nrf2 is disrupted, allowing Nrf2 to accumulate and translocate into the nucleus. There, Nrf2 forms heterodimers with small Maf (sMAF) proteins and binds to antioxidant response elements (AREs) in the promoter regions of target genes, thereby activating the transcription of antioxidant and cytoprotective genes, such as NQO1, HO-1, GCL, GST, and GPx.

In response to oxidative stress, cells activate a range of protective mechanisms. A central regulator of these defenses is nuclear factor erythroid 2-related factor 2 (Nrf2), a transcription factor that plays a critical role in maintaining redox homeostasis [83,84]. Nrf2 is abundantly expressed in oxygen-consuming organs, including the heart, liver, brain, kidneys, skeletal muscle, and blood vessels [84]. It is composed of 605 amino acids and contains 7 functional domains, referred to as Nrf2-ECH (erythroid cell-derived protein with CNC homology) (Neh) domains 1–7, which are essential for its stability and transcriptional regulation [85,86,87]. Among these, Neh1, Neh3, Neh4, and Neh5 are involved in transcriptional activation, whereas Neh2, Neh6, and Neh7 serve as negative regulatory domains [88]. Under basal conditions, Nrf2 is retained in the cytoplasm by Kelch-like ECH-associated protein 1 (Keap1). Keap1 mediates the polyubiquitination of Nrf2 through its interaction with a Cullin-3 (Cul3)-based E3 ubiquitin ligase complex, targeting Nrf2 for proteasomal degradation. This tight regulation ensures low basal levels of Nrf2 activity under normal physiological conditions [89]. Upon exposure to oxidative or electrophilic stress, cysteine residues on Keap1 are chemically modified, disrupting the Keap1-Nrf2 interaction. Consequently, Nrf2 escapes ubiquitination, accumulates in the cytoplasm, and translocates to the nucleus. There, it forms heterodimers with small musculoaponeurotic fibrosarcoma (sMAF) proteins and binds to antioxidant response elements (AREs) in the promoter regions of target genes [90]. This transcriptional program induces a wide array of cytoprotective genes involved in antioxidant defense, detoxification, and mitochondrial protection, including heme oxygenase-1 (HO-1), NAD(P)H quinone oxidoreductase 1 (NQO1), glutathione-S-transferase (GST), glutamate-cysteine ligase (GCL), glutathione peroxidase (GPx), thioredoxin (TXN), thioredoxin reductase (TXNRD1), and reduced glutathione (GSH) synthesis enzymes (Figure 3) [90,91,92]. Activation of the Nrf2-ARE pathway increases cellular energy and redox potential, thereby reducing oxidative damage [91]. Nrf2 can also bind to NF-κB target genes and repress their transcription [93]. These transcription factors exhibit a dynamic and antagonistic crosstalk, where activation of one often leads to suppression of the other [94]. NF-κB can inhibit Nrf2 activity by competing for shared transcriptional coactivators like CBP (CREB-binding protein) and recruiting HDAC3, which represses Nrf2-driven antioxidant gene expression [94,95]. Conversely, Nrf2 activation reduces intracellular ROS, stabilizes the NF-κB inhibitor IκB-α, and induces the expression of antioxidant and cytoprotective genes such as HO-1. Nrf2 can also directly repress transcription of NF-κB target genes, independently of the presence of ARE or NF-κB binding sequences, likely through interactions with specific binding partners [96,97]. Loss of Nrf2 exacerbates NF-κB signaling, leading to increased production of pro-inflammatory cytokines and enhanced inflammatory damage [93]. This reciprocal regulation is particularly important in ischemia–reperfusion injury, where excessive oxidative stress and inflammation synergistically contribute to tissue damage.

Nrf2 activity can also be regulated through Keap1-independent mechanisms. One such pathway involves glycogen synthase kinase-3β (GSK-3β), which phosphorylates Nrf2 at specific serine residues (Ser334–338), generating a recognition motif for β-transducin repeat-containing protein (β-TrCP). β-TrCP, in conjunction with a Cullin-1 (Cul1)/Rbx1-based E3 ubiquitin ligase complex, targets Nrf2 for proteasomal degradation independently of Keap1. This alternative regulatory mechanism provides an additional layer of control, allowing for precise modulation of Nrf2 activity under varying cellular conditions [98,99]. Importantly, the transcriptional and post-translational regulation of Nrf2 is influenced by epigenetic mechanisms. Among these, histone acetylation and deacetylation are of particular significance. Acetylation of histones, mediated by HATs [92,100,101], such as acetyltransferase p300 and its paralog CBP, loosens the chromatin structure, promoting gene transcription. These enzymes can also directly acetylate Nrf2 itself, particularly in its transactivation domain, enhancing its DNA-binding affinity, stability, and transcriptional activity [102,103].

Conversely, HDACs remove acetyl groups from both histones and non-histone proteins, including Nrf2. Specific HDACs have been shown to modulate Nrf2 function, either by deacetylating Nrf2 or by influencing the transcription of its downstream targets. For instance, HDAC3 has been shown to suppress Nrf2 activity in various models of ischemia. Inhibition of HDAC3 leads to increased nuclear translocation of Nrf2 and enhanced expression of its downstream antioxidant genes, such as HO-1 and NAD(P)H NQO1. This suggests that HDAC3 acts as a negative regulator of Nrf2 under ischemic conditions [103,104]. Moreover, sirtuin 1 (SIRT1), a cytoplasmic sirtuin (class III HDAC), has been shown to bind and deacetylate Nrf2. Deacetylation of Nrf2 results in its instability, shortened half-life, and decreased cellular and nuclear levels. The diminished nuclear accumulation of Nrf2 is thus associated with reduced transcription of its target genes [102,105]. Beyond histone modification, HDACs also target a wide array of non-histone substrates. These include transcription factors and regulatory proteins such as p53, NF-κB (p65 subunit), p73, and Ku70, which play essential roles in apoptosis, inflammation, DNA repair, and metabolism. Through the deacetylation of these proteins, HDACs can modulate numerous cellular pathways relevant to ischemic injury [101]. For instance, two members of the class III HDACs, SIRT1 and SIRT3, deacetylate the transcription factors p73 and Ku70, respectively [101,106,107]. For more information on HDACs, effects on Nrf2, and their mechanisms of action, see Table 1.

In models of retinal and cerebral ischemia–reperfusion injury, HDAC inhibitors (HDACis), such as TSA, have demonstrated significant neuroprotective effects. These compounds enhance Nrf2 nuclear translocation, acetylation, and ARE-driven gene expression, thereby mitigating oxidative stress and neuronal apoptosis. Notably, in Nrf2-deficient animals, the protective effects of HDACis are markedly diminished, reinforcing the indispensable role of Nrf2 in mediating the benefits of epigenetic therapies [120].

Cerebral ischemia and ischemia–reperfusion injury remain significant clinical challenges, with limited effective therapeutic options. The complex interplay between HDACs, HATs, and Nrf2 represents a promising therapeutic target for ischemic stroke and other oxidative stress-related conditions. On one hand, enhancing Nrf2 activity through HAT-mediated acetylation or pharmacological HDAC inhibition could bolster endogenous antioxidant defenses. On the other, fine-tuning this pathway requires an in-depth understanding of cell-type specificity, intervention timing, and the balance between pro-survival and pro-death signaling [100,101]. Targeting these pathways, particularly through HDAC inhibition and HAT activation, holds great promise for the development of neuroprotective therapies in ischemic brain injury and beyond.

## 6. HDACs in Neuronal Survival and Apoptosis

Neuroprotection in ischemic stroke aims to prevent irreversible neuronal damage resulting from disrupted cerebral blood flow. At the molecular level, ischemic injury triggers a cascade of events including excitotoxicity, calcium overload, oxidative stress, inflammation, and apoptotic signaling. Notably, experimental models have shown that HDAC3, HDAC6, and HDAC11 exhibit increased expression in the early stages following ischemic insult, suggesting their contribution to the pathophysiology of stroke [101]. These isoforms modulate neuronal viability through both epigenetic and non-epigenetic mechanisms. HDAC3, a class I isoform, has been shown to mediate neuronal death through pro-apoptotic signaling. HDAC3 has neurotoxic effects in neurons, regulated by the Akt and GSK3β pathways, with GSK3β-mediated phosphorylation enhancing HDAC3 activity [121]. HDAC3 expression increases during early phases of experimental stroke, contributing to neurotoxicity, as described in the second and third sections. In ischemic models, knockdown of HDAC3 improves cortical neuron survival under OGD conditions, indicating its potential as a therapeutic target [122]. Moreover, HDAC3 has been implicated in repressing anti-apoptotic gene expression and facilitating DNA damage responses that exacerbate injury [123]. Studies have shown that HDAC3 deletion or inhibition can protect against DNA damage in non-cycling cells and reduce retinal ganglion cell death following optic nerve injury [124,125]. HDAC3 also plays a role in neuroprotective recovery following brain injury. Furthermore, pharmacological inhibition of HDAC3 has shown promise in mitigating behavioral and neuroimmune deficits in various brain disorders, offering potential therapeutic strategies for neurological conditions [126].

HDAC6, a cytoplasmic class IIb isoform, regulates protein homeostasis and intracellular transport via its deacetylation of non-histone substrates such as α-tubulin [127]. During ischemia, HDAC6 is upregulated, promoting axonal degeneration and mitochondrial dysfunction [114]. Pharmacological inhibition of HDAC6 enhances tubulin acetylation, preserves mitochondrial integrity, and reduces ROS levels, thereby improving neuronal resilience to ischemic stress [114]. HDAC6 also facilitates the formation of protein aggregates and supports their clearance via autophagy, along with the degradation of dysfunctional mitochondria. These functions may offer protection against neurodegeneration by promoting cellular quality control mechanisms [128]. Thus, the overall impact of HDAC6 on neuronal health likely depends on a delicate balance between its roles in promoting axonal damage and supporting the removal of toxic cellular components [129]. Selective HDAC6 inhibitors, such as Tubastatin A (TubA), have shown neuroprotective effects without the toxicity observed with pan-HDAC inhibitors, making them attractive candidates for clinical development [130,131].

Beyond isoform-specific effects, HDACis have demonstrated strong neuroprotective properties in ischemic stroke models. Compounds such as valproic acid (VPA), sodium butyrate (SB), and TSA have been shown to reduce infarct size, attenuate microglial activation, and improve sensory and motor outcomes in rodent models, even when administered several hours post-occlusion [132,133]. These agents exert their effects by modulating key pathways involved in oxidative stress, inflammation, and apoptosis. For example, HDACi treatment leads to activation of the Nrf2 pathway, upregulating antioxidant enzymes such as heme oxygenase-1 (HO-1) and promoting cellular redox homeostasis [120] (more can be found in Section 5). HAT inhibitors have shown neuroprotective effects in various neurodegenerative models by modulating Tumor Protein P53 (p53) acetylation and its downstream targets. HDACis prevent DNA damage-induced neuronal apoptosis by altering p53 acetylation at lysine residues K381 and K382, which reduces its binding to the p53 upregulated modulator of apoptosis (PUMA) gene promoter and subsequent apoptosis induction [134]. This neuroprotective effect is observed in both p53-dependent and p53-independent pathways, with HDACis suppressing PUMA expression and preventing caspase activation [135]. Another study demonstrated that depletion of HDAC1 and HDAC2 promotes retinal ganglion cell survival after injury by inhibiting the p53-PUMA apoptosis pathway [136]. One key advantage of HDACis lies in their selectivity—targeted inhibition avoids the systemic side effects typically associated with broad-spectrum HDAC blockade, thus enhancing their therapeutic viability [137,138]. Despite these promising findings, the dualistic nature of HDAC activity necessitates caution. For instance, while HDAC1 and HDAC2 are widely recognized as neuroprotective and indispensable for proper neuronal development, their function is highly context-dependent and may become neurotoxic under specific pathological conditions [139]. During normal neurodevelopment, HDAC1 and HDAC2 redundantly regulate neuronal specification and differentiation. Deletion of either isoform alone produces no overt phenotype. However, combined ablation results in profound structural and functional abnormalities in the brain, including disrupted hippocampal and cerebellar architecture, due to impaired maturation of neuronal precursors and excessive apoptotic cell death [139,140,141]. Beyond development, HDAC1 contributes to neuronal survival in mature neurons through its role in the p130–E2F4–Suv39H1–HDAC1 complex (where p130 is a retinoblastoma-like protein 2, E2F4 is a member of the E2F transcription factor family, and Suv39H1 is histone-lysine N-methyltransferase), which represses the transcription of pro-apoptotic genes [140]. Disruption of this complex by apoptotic stimuli leads to de-repression of death-promoting genes and consequent neuronal degeneration [142]. However, HDAC1 can also contribute to neurotoxicity when aberrantly activated by the p25/Cdk5 complex (where p25 is a truncated activator of cyclin-dependent kinase 5), leading to DNA damage and cell-cycle reentry in post-mitotic neurons [143]. Moreover, in Alzheimer’s disease models, HDAC1 deregulation by the p25/Cdk5 complex leads to aberrant cell-cycle reentry and accumulation of DNA damage, culminating in neuronal loss. Restoring HDAC1 function in this context, either by enhancing its enzymatic activity or re-establishing proper complex formation, can prevent DNA damage and protect against neurotoxicity [143,144]. HDAC1’s neuroprotective or neurotoxic role appears to depend on its binding partners. When associated with histone deacetylase-related protein (HDRP), a truncated, catalytically inactive HDAC9 variant, HDAC1 promotes neuronal survival. This protective effect is lost when HDAC1 forms a complex with HDAC3 instead [145]. Therefore, HDAC1 functions as a molecular switch, with its impact on neuronal fate being governed not only by its expression level or catalytic activity but also by the specific protein complexes it engages in. Similarly, HDAC4 displays isoform-specific divergence; its cytoplasmic retention promotes survival, but nuclear translocation triggers apoptosis in cerebellar granule neurons [146].

In the nervous system, HDACs have gained attention for their role in neuroprotection, especially by influencing the expression of BDNF, a protein essential for neuronal survival, synaptic plasticity, and cognitive function. HDAC inhibitors have been shown to significantly increase BDNF expression. HDAC3 has been identified as a key negative regulator of genes involved in synaptic plasticity and neuronal survival, such as Bdnf and Npas4 (Neuronal PAS domain protein 4). Research has shown that HDAC3 binds to the promoters of these neuroprotective genes—especially in neurons that are vulnerable or primed to die—resulting in their transcriptional repression. This silencing limits the expression of critical survival factors. By inhibiting HDAC3, it may be possible to restore Bdnf and Npas4 expression, thereby enhancing neuronal resilience and offering protection against neurodegenerative processes [147]. Interestingly, different HDAC classes regulate BDNF expression in distinct ways. Inhibition of class II HDACs, such as HDAC4 and HDAC5, results in a rapid increase in BDNF mRNA levels, while inhibition of class I HDACs leads to a delayed but sustained increase. This suggests that class II HDACs directly repress BDNF promoter IV activity, and selective inhibition of these enzymes may offer therapeutic potential for neurological disorders by enhancing BDNF-mediated signaling [148]. In models of traumatic brain injury (TBI), HDAC inhibition has been associated with elevated BDNF levels, enhanced neuronal rewiring, and improved motor recovery. Specifically, inhibition of HDAC2 increased H4K5 acetylation at BDNF promoters and elevated BDNF mRNA expression, supporting synaptic plasticity and functional regeneration [149]. Studies have shown that HDACis, such as SB and TSA, increase BDNF and GDNF transcription in astrocytes. This glial-derived support contributes to dopaminergic neuron survival and presents a novel mechanism for treating psychiatric and neurodegenerative conditions [150]. Furthermore, hydroxamic acid-based HDAC inhibitors (hb-HDACis) have been identified as potent enhancers of BDNF expression at both the mRNA and protein levels. These compounds also promote neurite outgrowth in human neural progenitor cells, suggesting their potential for supporting neurogenesis and recovery from neuronal loss [151]. Interestingly, intact HDAC activity is also required for BDNF to exert its full synaptic effects. BDNF-induced increases in dendritic spine density and excitatory neurotransmitter release in CA1 pyramidal neurons depend on balanced histone acetylation and deacetylation. This highlights that while HDAC inhibition can enhance BDNF expression, a certain level of HDAC activity is still necessary for BDNF signaling and synaptic function [152]. The relationship between HDACs, BDNF, and neurodegenerative diseases such as Alzheimer’s disease (AD) is also significant. The apolipoprotein E epsilon 4 (ApoE4) allele, a major genetic risk factor for AD, promotes nuclear translocation of HDACs, leading to suppression of BDNF expression. In contrast, the protective apolipoprotein E3 (ApoE3) allele increases histone acetylation and enhances BDNF transcription. Similarly, amyloid-beta (Aβ) oligomers—implicated in AD pathology—reduce BDNF levels through HDAC-mediated mechanisms. Importantly, blocking low-density lipoprotein receptor-related protein 1 (LRP-1) or activating epsilon protein kinase C epsilon (PKCε) can reverse these effects, suggesting potential targets for restoring BDNF expression in AD [153,154]. In ischemic and hypoxic conditions, HDAC inhibitors have shown promise by upregulating BDNF expression through increased histone acetylation. For example, compound 13 has been reported to enhance BDNF levels and cell viability by increasing acetylation of histone residues histone H3 lysine 14 (H3K14) and histone H4 lysine 5 (H4K5), thereby stimulating BDNF promoter activity. This provides a mechanistic basis for the use of HDACis in preventing neuronal damage due to insufficient blood or oxygen supply [155].

One of the most prominent HATs involved in ischemic neuroprotection is the CREB-binding protein (CBP), a transcriptional coactivator with intrinsic HAT activity. CBP plays a pivotal role in regulating neuronal survival during ischemia through the acetylation of both histone and non-histone proteins [156,157]. Experimental studies have shown that CBP-mediated histone acetylation is essential for neuronal resistance to OGD and that CBP deficiency significantly increases vulnerability to ischemic injury [157]. CBP activity is dynamically regulated during stress. Under physiological conditions, transcriptional activators such as Zta (Z Epstein–Barr virus transcriptional activator), nuclear factor erythroid 2 (NF-E2), and CCAAT/enhancer-binding protein alpha (C/EBPα) enhance CBP’s HAT activity and promote acetylation at promoters of neuroprotective genes [158]. However, in ischemic and neurodegenerative conditions, CBP becomes a substrate for calpains and caspases, particularly caspase-6, leading to its proteolytic cleavage and inactivation. This process contributes to the global reduction in histone acetylation observed during neuronal apoptosis and may exacerbate neuronal death after stroke [45]. IPC, a process by which sublethal ischemia enhances resistance to subsequent ischemic events has been shown to increase CBP expression and recruitment to the promoters of protective genes such as gelsolin, accompanied by elevated levels of histone acetylation [157]. Blocking CBP’s HAT activity abolishes the protective effects of IPC, underscoring the critical role of CBP-driven histone acetylation in this endogenous neuroprotective mechanism [159]. Furthermore, the therapeutic modulation of histone acetylation through external interventions has gained increasing attention as a strategy to enhance endogenous neuroprotective mechanisms. One such intervention is electroacupuncture (EA), a modern adaptation of traditional acupuncture that combines needle insertion at specific acupoints with the application of low-frequency electrical stimulation. EA has been shown to exert beneficial effects on the central nervous system, particularly in models of cerebral ischemia. Recent studies have demonstrated that EA can promote histone acetylation at the promoter regions of genes involved in neuronal survival and apoptosis regulation, such as Bcl-2 and caspase-3. This epigenetic modulation enhances the expression of anti-apoptotic factors while suppressing pro-apoptotic genes, contributing to reduced neuronal death and improved functional recovery after stroke. In rat models, EA enhances histone H3 at lysine 9 (H3K9) and lysine 27 (H3K27) acetylation at the Bcl-2 promoter while reducing acetylation at the caspase-3 promoter, thereby promoting anti-apoptotic gene expression and suppressing pro-apoptotic pathways [160]. EA also regulates histone acetylation at MMP-9 and TIMP-2 promoters, preserving blood–brain barrier. In addition, EA modulates the acetylation of histone H4 at lysine 16 (H4K16) and promotes autophagy through the SIRT1–beclin1 pathway, contributing to the reduction in ischemia–reperfusion injury [161]. The importance of HAT activity in neuroprotection is further reinforced by evidence that pharmacological inhibition of CBP or other HATs abolishes these beneficial effects, leading to exacerbated neuronal damage. This indicates that HATs do not merely counterbalance HDACs but play an active, indispensable role in regulating ischemia-responsive gene networks. Conversely, in many neurodegenerative conditions, including ischemic brain injury, reduction in HAT function and CBP levels correlates with disease progression, highlighting the pathological consequences of impaired acetylation homeostasis [162]. Therefore, HATs such as CBP are central to the neuroepigenetic response to ischemia. Their capacity to regulate gene expression, chromatin structure, and neuronal resilience places them as promising therapeutic targets alongside HDACis [163,164,165,166]. Therapies aimed at enhancing HAT activity or mimicking its downstream effects—possibly in combination with HDAC inhibition —may provide synergistic benefits in reducing infarct size, promoting functional recovery, and preventing long-term neurodegeneration [165].

## 7. HDACs in Glial, Endothelial, and Neurovascular Integrity

Ischemic stroke leads not only to neuronal loss but also to profound vascular dysfunction, particularly involving the BBB. HDACs, especially via epigenetic mechanisms, have been increasingly recognized as critical modulators of vascular stability and BBB integrity in this context. HDACs modulate vascular cell functions, including endothelial cell and smooth muscle cell proliferation, migration, and apoptosis [167,168]. Among the HDAC isoforms, HDAC9 has emerged as a key contributor to endothelial injury and blood–brain barrier (BBB) disruption in the context of cerebral ischemia/reperfusion injury [64]. In particular, increased HDAC9 levels have been observed in the ipsilesional cortex after MCAO in mice [169]. The protective mechanisms involve suppression of the inhibitor of nuclear factor kappa B alpha (IκBα)/NF-κB and MAPK signaling pathways, as well as regulation of the microRNA-20a (miR-20a)/neuronal differentiation 1 (NeuroD1) axis [65,170]. Silencing HDAC9 has been shown to reduce infarct size, suppress pro-inflammatory cytokine expression (e.g., IL-1β, TNF-α, IL-6), and improve neurological function, underscoring its potential as a therapeutic target in ischemic stroke [65,170]. Recent research has revealed HDAC9’s involvement in the post-translational regulation of key transcription factors, notably HIF-1 and specificity protein 1 (Sp1). In experimental stroke models, HDAC9 physically interacts with these transcription factors and enzymatically deacetylates them, altering their transcriptional activity. Deacetylation of HIF-1 leads to upregulation of transferrin receptor 1 (TfR1), which increases cellular iron uptake. Simultaneously, HDAC9-mediated deacetylation of Sp1 results in the downregulation of glutathione peroxidase 4 (GPX4), a critical enzyme involved in protecting cells from lipid peroxidation and ferroptosis. The combination of increased TfR1 expression and reduced GPX4 activity promotes iron accumulation and oxidative stress, ultimately driving neuronal ferroptosis in the ischemic brain [112]. Finally, HDAC11 has also been implicated in immune regulation, particularly in neutrophils and T cells. It acts as a negative regulator of the anti-inflammatory cytokine IL-10, influencing the magnitude and duration of inflammatory responses during ischemic injury [171].

HDACis have demonstrated potent vascular protective effects in various ischemic models. These agents mitigate ischemia-induced brain damage by targeting multiple mechanisms, including excitotoxicity, oxidative stress, apoptosis, and neuroinflammation [133,172]. Among them, VPA has been shown to attenuate ischemia-induced BBB disruption in both transient and permanent MCAO models. VPA reduces MMP9 expression, inhibits NF-κB nuclear translocation, and prevents tight junction degradation, including the loss of claudin-5 and occludin, thereby preserving BBB integrity [173,174]. These effects are mediated via HDAC inhibition and further supported by increased expression of protective proteins such as HSP70 and Bcl-2 [174,175]. In addition to reducing infarct volume and neurological deficits, VPA also preserves vascular architecture and suppresses neural apoptosis through activation of the HSP70/protein kinase B (Akt) and HSP70/matrix metalloproteinase pathways, highlighting its dual action on both neural and vascular compartments [176]. Similar vascular protective effects have been observed with other HDACis, such as SB and TSA, which also suppress ischemia-induced inflammation and MMP activity [132]. These findings underscore the broader impact of HDAC regulation on the entire neurovascular unit, extending beyond neurons to include glial and endothelial cell populations. Moreover, HDACis are effective not only in acute injury models but also in aged animals, where they preserve axonal structure and function during ischemia. Studies demonstrate that HDACis preserve axonal function and structure in both young and aged animal models by conserving ATP, reducing excitotoxicity, and maintaining mitochondrial integrity [47]. HDACis remain effective even when administered after injury, improving functional recovery and attenuating tissue damage in models of TBI [177]. Studies have shown that HDACis significantly reduce infarct size and improve functional outcomes in preclinical models of stroke and myocardial ischemia. These protective effects are mediated through multiple mechanisms, including the modulation of gene expression programs related to hypoxia, cell death, and vascular permeability [114]. Furthermore, HDACs have been implicated in cardiovascular disease and atherosclerosis, suggesting that shared epigenetic mechanisms may regulate both cerebral and systemic vascular responses [167,178].

## 8. HDACs as Therapeutic Targets in Ischemia

HDACis have emerged as promising therapeutic agents for ischemic injuries in both cerebral and myocardial tissues. In models of cerebral ischemia, HDACis confer robust neuroprotection by modulating a range of pathological processes, including excitotoxicity, oxidative stress, inflammation, endoplasmic reticulum stress, and BBB disruption. Moreover, HDACis promote beneficial responses such as angiogenesis, neurogenesis, and stem cell migration, leading to reduced infarct volumes and improved functional recovery even when treatment is delayed [172].

HDACis have demonstrated significant neuroprotective effects in models of cerebral ischemia. HDAC inhibitors fall into four major classes: hydroxamic acids (e.g., TSA, SAHA), cyclic ketones (e.g., trapoxins), short-chain fatty acids (e.g., SB, VA), and benzamides. Several of these compounds have shown efficacy in preclinical ischemic stroke models. Studies using rat models of stroke have consistently demonstrated the neuroprotective effects of HDACis in ischemic brain injury [179,180]. The pan-HDAC inhibitor is a class of drugs that can inhibit all types of histone deacetylases (SAHA has been shown to enhance neuroplasticity in surviving neurons within the peri-infarct area) [181]. When administered during early reperfusion in hypertensive rats following tMCAO, SAHA significantly reduced cerebral infarct volume, attenuated microglial activation, and preserved the BBB, thereby exerting potent cerebrovascular protective effects [182].

TSA, another pan-HDAC inhibitor from the hydroxamic acid class, has demonstrated similar protective effects. TSA increases histone H3 acetylation following permanent MCAO (pMCAO), resulting in significant improvements in motor, sensory, and reflex functions in rats. Furthermore, TSA mitigates ischemic stroke damage by reducing autophagy and correcting lysosomal dysfunction in penumbral neurons [183].

Another HDAC inhibitor, the short-chain fatty acid sodium butyrate, also shows neuroprotective properties in ischemic stroke models. Intranasal administration of sodium butyrate one hour after MCAO significantly ameliorated apoptosis in rats [184]. Additionally, it decreased levels of GFAP in serum and improved BBB integrity [185]. Sodium butyrate modulates microglial behavior by promoting a shift from pro-inflammatory to anti-inflammatory phenotypes under LPS stimulation, thereby attenuating microglia-mediated neuroinflammation [186]. It also inhibits systemic inflammatory responses by modulating regulatory T cells and associated inflammatory pathways. These actions contribute to its neuroprotective effects in diabetic stroke models [187]. Valproic acid, another short-chain fatty acid HDAC inhibitor, has also demonstrated neuroprotection in ischemic injury. In vivo studies report that VPA treatment reduces the number of TUNEL-positive cells in the ischemic border zone and mitigates ischemia–reperfusion injury by suppressing oxidative stress and inflammation [188]. Although low-dose VPA does not significantly reduce infarct volume, it promotes anti-inflammatory M2 microglial polarization in the peri-infarct cortex and gradually decreases the number of activated microglia over time [189]. VPA appears to modulate microglial morphology through HDAC inhibition and suppression of galectin-3 production. In both in vitro and in vivo studies, VPA has been shown to induce the expression of heat shock protein 70.1B, inhibit glial scar formation, and protect against neuronal damage during stroke recovery [189]. In BV-2 cells induced by OGD, VPA reduces apoptosis and microglia injury [190]. Similarly, in a gerbil model of transient global cerebral ischemia, VPA treatment reduced pyroptosis and hippocampal damage, highlighting its broad efficacy in ischemia–reperfusion injury. These neuroprotective effects may be facilitated by VPA’s ability to penetrate the BBB [173].

Li et al. demonstrated that in a mouse model of cerebral ischemia/reperfusion injury, HDAC6 expression was significantly upregulated. Silencing HDAC6 using shRNA led to marked reductions in neurological damage, infarct size, and oxidative stress markers, including 3-nitrotyrosine (3-NT), 4-hydroxynonenal (4-HNE), and 8-hydroxy-2′-deoxyguanosine (8-OHdG). The observed neuroprotective effects were attributed to the activation of the Nrf2/HO-1 signaling pathway, which plays a critical role in regulating antioxidant responses. These findings suggest that HDAC6 inhibition mitigates I/RI-induced oxidative stress and enhances endogenous antioxidant defenses, positioning HDAC6 as a potential therapeutic target in ischemic stroke [191].

Consistent with these findings, isoform-selective inhibition of HDAC6 using TubA has shown significant therapeutic potential. TubA treatment in rats subjected to MCAO improved functional recovery, reduced infarct volume, and protected against neuronal cell death [192]. HDAC6 is known to deacetylate non-histone proteins, such as macrophage migration inhibitory factor (MIF), and its inhibition by TubA markedly increases MIF acetylation, a modification that contributes to its neuroprotective function in ischemic stroke models [193]. Moreover, other isoform-specific HDACis, such as MI-192—which selectively targets HDAC2 and HDAC3—have demonstrated neuroprotective effects as well. In a photothrombotic stroke (PTS) mouse model, MI-192 significantly reduced infarct core volume and apoptosis while partially restoring functional symmetry in forelimb use [194]. Sirtuins (SITRs), a family of NAD^+^-dependent deacetylases, have also been implicated in the response to cerebral ischemia. Sirtinol, a non-selective SIRT inhibitor that antagonizes SIRT1, has been shown to induce microglial activation under OGD/reoxygenation conditions and exacerbate ischemic injury [117,195]. In contrast, SIRT1 overexpression enhances SIRT3 activity through deacetylation, offering protection against ischemia–reperfusion-induced mitochondrial dysfunction and neuronal damage. However, the neuroprotective effects of SIRT1 are diminished when co-administered with the SIRT3 inhibitor 3-TYP, suggesting that the SIRT1–SIRT3 axis is essential for full neuroprotection [196]. Although high doses of SIRT3 inhibitors worsen ischemic injury, the off-target effects associated with SIRT1 modulation caution against direct SIRT3 inhibition. Strategies that enhance SIRT1 expression may be more effective in conferring neuroprotection [197]. In vivo, the use of SIRT2 inhibitors such as AK1 and AGK2 has resulted in reduced infarct size, improved neurological outcomes, and decreased apoptotic cell death in ischemic models [198]. These inhibitors also attenuate neuroinflammation and regulate microglial polarization [199,200]. AK7, another SIRT2 inhibitor, exerts dose-dependent neuroprotective effects in models of ischemia, likely through inhibition of P38 activation and modulation of microglial responses [201]. Recent research has focused on SIRT5 as a novel therapeutic target in ischemic stroke. The SIRT5 inhibitor MC3482 enhances the succinylation of annexin-A1, promoting its membrane localization and extracellular secretion. This mechanism alleviates microglia-induced neuroinflammation and improves long-term neurological function in stroke models [202]. Additionally, lentiviral overexpression of SIRT5 in both rat cerebral ischemia–reperfusion injury (CI/RI) models and H19-7 hippocampal neurons reduced neuronal damage and inhibited ferroptosis, further supporting its neuroprotective role [202]. A summary of HDACis in ischemia therapy is presented in Table 2.

In parallel, attention has turned to HATs, a class of enzymes that, in contrast to HDACs, catalyze the addition of acetyl groups to histone proteins, thereby promoting gene transcription. HATs are broadly categorized into two types: type A HATs, which function within the nucleus to acetylate histones in chromatin and facilitate transcriptional activation, and type B HATs, which operate in the cytoplasm to acetylate newly synthesized histones prior to their incorporation into chromatin [207]. HATs are involved in numerous biological processes, including gene expression regulation, DNA damage repair, and synaptic plasticity, and play essential roles in both the developing and mature brain [74,208].

Several pharmacological modulators of HAT activity have shown promise in neurological applications. Lys-CoA, one of the first HAT inhibitors developed, selectively targets p300 and PCAF, enhancing the precision of HAT inhibition [207]. Its ability to enhance synaptic plasticity in mice underscores its relevance to learning and memory [209]. Garcinol, a naturally derived polyisoprenylated benzophenone from Garcinia morella, is another potent HAT inhibitor with strong activity against p300 and PCAF. Notably, garcinol has demonstrated the capacity to reduce oxidative stress and inflammatory responses in both in vitro and in vivo models of ischemia/reperfusion (I/R) injury, underscoring its therapeutic potential for mitigating cerebral damage [210,211].

Small-molecule modulators targeting the HAT pathway have also gained attention for their neuroprotective properties. A485, a selective inhibitor of CBP/p300, has been shown to suppress the formation of lysine-lactylated (Kla) proteins, which play a role in the pathogenesis of IS. Treatment with A485 reduced neuronal death and glial cell activation, thereby minimizing brain injury in murine stroke models [212,213]. Conversely, activation of CBP/p300 has also shown beneficial effects. The small-molecule CBP/p300 activator TTK21, especially when conjugated to carbon spheres to form CSP-TTK21, exhibits potent neuroregenerative and cognitive-enhancing effects. This formulation efficiently crosses the blood–brain barrier without inducing toxicity and has been associated with enhanced neuroplasticity and long-term memory improvement, positioning it as a promising candidate for neurorestorative therapies following IS [212,214,215].

A growing body of research highlights the neuroprotective potential of various natural compounds in the context of ischemic stroke, primarily through their ability to modulate epigenetic mechanisms such as histone acetylation and sirtuin signaling. One of the most well-studied compounds is curcumin, which has been identified as a dual inhibitor of histone acetyltransferases and histone deacetylases [215,216], effectively reducing histone acetylation and influencing ischemic tolerance, while also upregulating SIRT1 expression to protect against brain injury [217]. Similarly, tea polyphenols, especially EGCG (epigallocatechin-3-gallate), help restore the balance between HATs and HDACs in inflammatory pathways [218]. Berberine exerts its neuroprotective effects by regulating a broad array of epigenetic factors and enhancing m6A methylation through METTL3 [219].

Resveratrol, a well-known SIRT1 activator, not only extends the window of ischemic tolerance but also modulates mitochondrial function and inflammatory signaling, with additional benefits observed when combined with HDACis [220]. A wide range of flavonoids, such as wogonin, apigenin, quercetin, icariside II, and trilobatin, have demonstrated the ability to activate different members of the sirtuin family (SIRT1–SIRT7), leading to reduced inflammation, oxidative stress, and neuronal damage, as well as improved cognitive function and post-stroke recovery [74,204,221]. Natural compounds derived from Astragalus membranaceus, including astragaloside IV and cycloastragenol, enhance SIRT1 and SIRT7 activity, contributing to neuroprotection and vascular repair [222,223]. Other bioactives like forsythoside B and pterostilbene further support brain recovery by modulating sirtuin signaling and HDAC expression [224].

Collectively, these findings suggest that natural compounds such as curcumin, polyphenols, and flavonoids exert multi-targeted epigenetic effects by modulating HATs, HDACs, and sirtuins, contributing to the regulation of inflammation, oxidative stress, and neuronal survival. These compounds represent a novel class of potential epigenetic therapeutics for the treatment of ischemic stroke, offering the advantages of low toxicity, natural origin, and broad-spectrum activity [74]. More detailed mechanisms and compound-specific information are provided in Table 3.

The therapeutic potential of HDAC and HAT inhibitors in ischemic stroke has gained increasing attention due to their roles in epigenetic regulation of neuroinflammation, oxidative stress, and neuronal survival. However, the clinical translation of these agents faces substantial challenges.

One of the primary limitations in developing HAT inhibitors is the structural and functional complexity of these enzymes. HATs often engage in intricate protein–protein interactions, and currently available inhibitors suffer from low potency and poor isoform selectivity, limiting their therapeutic application. Additionally, there is a notable gap between the in vitro efficacy of HAT inhibitors and their performance in in vivo or clinical settings [234]. Understanding the molecular underpinnings of HAT function may help guide the development of more effective and selective inhibitors. Similarly, while HDACis have demonstrated neuroprotective effects in various preclinical models, their clinical application is constrained by multiple factors. These include limited brain penetration, non-specificity across HDAC isoforms, and systemic toxicity—notably cardiac toxicity and low accumulation in solid tissues [235]. Moreover, the complex landscape of lysine post-translational modifications and variable activity among HDAC isoforms further complicate pharmacological targeting [236].

The biological diversity of HDAC isoforms also presents a challenge. Although pan-HDAC inhibitors like trichostatin A and sodium butyrate have shown effectiveness in reducing infarct size and enhancing functional recovery, the specific roles of individual HDACs in ischemia remain controversial [237]. Additionally, histone acetylation is significantly suppressed following ischemic injury, particularly at histones H3 and H4, leading to transcriptional repression. HDACis have been shown to reverse these effects, promoting neurogenesis, angiogenesis, and functional recovery after cerebral ischemia [238]. Despite these hurdles, HDAC inhibitors exhibit a favorable therapeutic window. Several compounds have proven effective even when administered hours after ischemic onset, which is especially promising for clinical stroke management where early intervention is not always feasible [172]. Furthermore, HDACis like valproic acid and sodium butyrate have demonstrated cardio- and neuroprotective effects in ischemia/reperfusion injury models, supporting their broader utility [239].

Looking forward, advances in isoform-selective HDACis and targeted delivery systems, such as nanoparticle-based carriers, are expected to improve drug efficacy while minimizing systemic toxicity. Additionally, combination therapies that integrate HDAC or HAT inhibition with anti-inflammatory or antioxidant agents may further enhance therapeutic outcomes. The growing interest in precision medicine and biomarker-based approaches also holds promise for tailoring epigenetic therapies to individual patients, improving safety and efficacy profiles.

In summary, although HDAC and HAT inhibitors present promising avenues for ischemic stroke therapy, substantial challenges related to selectivity, delivery, pharmacokinetics, and toxicity must be addressed. Continued research focused on the molecular mechanisms, isoform-specific functions, and innovative delivery strategies will be crucial to unlocking their full clinical potential.

## 9. Conclusions

Epigenetic regulation has emerged as a pivotal mechanism in shaping the cellular response to ischemic injury, particularly through the dynamic interplay between histone deacetylases (HDACs) and histone acetyltransferases (HATs). HDACs, especially isoforms such as HDAC1, HDAC2, HDAC3, and HDAC6, are consistently implicated in promoting neuroinflammation by enhancing pro-inflammatory gene expression in microglia, astrocytes, Müller glia, and infiltrating immune cells. In contrast, HATs such as CBP/p300 and PCAF often exert protective effects by promoting transcription of anti-inflammatory and neuroprotective genes through increased histone acetylation. This dual role underscores the potential of HDAC inhibition and HAT activation as complementary therapeutic strategies. HDAC inhibitors have shown promising results in reducing infarct volume, restoring homeostatic glial functions, and limiting inflammatory cascades. The greater emphasis on HDACs in this review reflects the current state of the literature, as research on HATs remains comparatively limited. This imbalance is due to the broader availability of data and mechanistic insights pertaining to HDACs. Meanwhile, enhancing HAT activity—although less explored—offers a compelling avenue to support neuronal survival and immune resolution following ischemia.

Future research must aim to clarify isoform-specific functions of HDACs and HATs across different CNS cell types and ischemic stages, develop cell-targeted or brain-penetrant epigenetic modulators with minimal off-target effects, explore combinatorial therapies that leverage HDAC inhibitors with HAT activators or immune modulators, and investigate long-term epigenetic reprogramming effects on neuronal regeneration, glial scar formation, and post-stroke cognitive outcomes. Ultimately, understanding the context-dependent roles of HDACs and HATs will enable the design of precision epigenetic therapies that not only dampen harmful inflammation but also facilitate repair and recovery in ischemic neurodegeneration.

## Figures and Tables

**Table 1:** Epigenetic regulation of Nrf2 by HDAC isoforms in different cell types under ischemic conditions.

HDAC	Class	Effect on Nrf2	Mechanism	Cell Type Specificity	Effect in Ischemia	References
HDAC2	Class I	Enhances Nrf2 activity	Deacetylates Nrf2, stabilizing it and promoting antioxidant gene expression; reduced HDAC2 impairs Nrf2 activation, increasing oxidative stress sensitivity	Neurons, astrocytes, inner retinal layers, microglia	Selective reduction in HDAC2 significantly protects the retina from ischemic injury.	[18,108]
HDAC3	Class I	Suppresses Nrf2 activity	Binds and deacetylates Nrf2, leading to instability, reduced half-life, and lower nuclear accumulation → decreased gene transcription	Myeloid cells, neurons, astrocytes, microglia, macrophages	Deletion of HDAC3 in myeloid cells promotes efferocytosis and reduces ischemic retinal injury.	[18,60,103,109]
HDAC5	Class IIa	Suppresses Nrf2 via catalytic domain	Catalytic activity decreases mitochondrial ROS, thereby reducing Nrf2 activation; inhibition or knockdown increases Nrf2-mediated transcription	Neurons	Suppresses Nrf2-mediated protection by decreasing mitochondrial ROS. Inhibition improves outcomes post-ischemia through histone re-acetylation and BMP7 (bone morphogenetic protein 7) induction.	[50,110,111]
HDAC9	Class IIa	No effect on Nrf2 via catalytic activity. Indirect association with oxidative stress via NF-κB signaling, but no direct catalytic regulation of Nrf2.	Catalytic domain not required for hypertrophy regulation; knockdown does not stimulate Nrf2 transcription	Neurons, macrophages, endothelial cells, microglia	HDAC9 deletion or silencing led to smaller infarct volumes, improved neurological outcomes, and suppression of systemic and neuroinflammatory cytokines.	[64,65,111,112]
HDAC6	Class IIb	Dual role—context-dependent activation or suppression of Nrf2	Inhibition can enhance Nrf2 signaling and antioxidant gene expression; in some models, it suppresses Nrf2 activity	Astrocytes, microglia	Inhibition of HDAC6 can promote Nrf2 nuclear translocation in astrocytes and microglia under oxidative stress, though outcomes vary depending on injury model.	[113,114]
SIRT2	Class III (Sirtuin)	Suppresses Nrf2	Binds and deacetylates Nrf2, leading to instability, reduced half-life, and lower nuclear accumulation → decreased gene transcription	Microglia, neurons	Impairs Nrf2 stability and antioxidant defenses. Knockout or inhibition of SIRT2 protects against cerebral and cardiac ischemic injury.	[102,105,115,116]
SIRT1	Class III (Sirtuin)	Indirect effect. SIRT1 modulates oxidative stress via deacetylation of transcription factors involved in Nrf2-linked pathways (e.g., p53, NF-κB).	Deacetylates non-histone proteins (e.g., p73), influencing physiological processes (e.g., apoptosis, metabolism)	Neurons, endothelial cells	Provides neuroprotection indirectly through modulation of apoptosis and inflammation pathways (e.g., p53, NF-κB), with antioxidant effects contributing to improved ischemic outcomes.	[101,117]
SIRT3	Class III (Sirtuin)	Indirect effect	Deacetylates Ku70; impacts on Nrf2 not directly shown but involved in oxidative stress pathways	Mitochondria, neurons, cardiomyocytes	Reduces oxidative stress and promotes autophagy via AMPK-mTOR pathway. Indirectly supports Nrf2-related antioxidant defenses during cerebral and cardiac ischemia.	[101,118,119]

**Table 2:** HDAC inhibitors in ischemia therapy.

Inhibitor	Target	Effects	Model	References
SAHA	Pan-HDAC	Enhances neuroplasticity, reduces infarct volume, attenuates microglial activation, preserves BBB	tMCAO in hypertensive rats	[181,182,183]
TSA	Pan-HDAC	Increases histone H3 acetylation, improves sensorimotor function, reduces autophagy, activates Nrf2 pathway	pMCAO rats	[120,184,203]
Sodium butyrate	HDAC Class I and IIa	Reduces apoptosis, preserves BBB, promotes anti-inflammatory microglial phenotype, regulates T cells	MCAO in rats, LPS-stimulated microglia	[185,186,187]
VPA	HDAC Class I and II	Reduces apoptosis, promotes M2 polarization, inhibits oxidative stress, induces Hsp70.1B, reduces glial scar formation	MCAO, OGD in vitro, global ischemia in gerbils	[188,190,204,205]
HDAC6 shRNA	HDAC6	Reduces infarct size, oxidative stress, activates Nrf2/HO-1 pathway	Mouse I/R model	[191]
TubA	HDAC6	Improves recovery, reduces infarct, increases MIF acetylation	MCAO rats	[192,193]
MI-192	HDAC2, HDAC3	Reduces infarct and apoptosis, improves functional recovery, SIRT2 inhibitors	PTS mouse model	[194]
Sirtinol	SIRT1	Exacerbates injury, increases microglial activation	OGD/reoxygenation	[117,195]
AK1/AGK2	SIRT2	Reduce infarct, improve neurological outcome, decrease apoptosis, modulate microglia	Ischemia models	[198,199,200]
AK7	SIRT2	Dose-dependent neuroprotection, inhibits p38, modulates microglia	Ischemia models	[201]
MC3482	SIRT5	Increases annexin-A1 succinylation, reduces neuroinflammation, improves function	Stroke models	[206]

**Table 3:** Natural epigenetic modulators in ischemic stroke therapy.

Compound	Target	Mechanism	Effects	References
Curcumin	HAT (p300/CBP), HDAC, SIRT1	Inhibits p300/CBP, reduces H3/H4 acetylation, inhibits HDACs, upregulates SIRT1	Neuroprotection, disrupts ischemic tolerance, reduces inflammation	[157,215,216,217]
Garcinol	HAT (p300, PCAF)	Inhibits HAT activity	Reduces oxidative stress and inflammation in ischemia/reperfusion models	[210]
EGCG	HAT (p300/CBP), HDAC3	Decreases p300/CBP binding, increases HDAC3 recruitment	Anti-inflammatory, neuroprotection	[218]
Berberine	HATs (CREBBP, EP300, HAT1), HDACs (SIRT3, HDAC5/9), DNMTs	Upregulates certain HATs/HDACs, downregulates HDAC2/8, DNMT1/3B; regulates m6A methylation via METTL3	Epigenetic regulation, METTL3-mediated astrocyte protection	[219]
Resveratrol	SIRT1, HDAC1/2	Activates SIRT1, improves mitochondrial function, modulates UCP2, Shh/Gli-1, cAMP/AMPK/SIRT1 axis; HDAC1/2 inhibition with derivatives	Mimics ischemic preconditioning, reduces oxidative stress, suppresses microglial activation	[225,226,227,228]
Wogonin	AMPK/SIRT1 pathway	Activates AMPK/SIRT1	Inhibits NLRP3 inflammasome, anti-inflammatory	[221]
Apigenin	HDACs	Decreases HDACs, restores histone acetylation	Improves cognitive outcomes post-MCAO	[229]
Quercetin	SIRT1	Activates SIRT1	Improves BBB integrity, reduces ROS	[230]
Icariside II	SIRT6	Activates SIRT6	Alleviates post-stroke depression via gut–brain axis	[204]
Trilobatin	SIRT3, SIRT6/7	Activates SIRT3, increases SIRT6/7	Promotes angiogenesis via SIRT7/VEGFA	[231,232]
Astragaloside IV/Cycloastragenol	SIRT1, SIRT7	Upregulates SIRT1 and SIRT7	Reduces inflammation, promotes vascular repair	[222,223,233]
Forsythoside B	SIRT1	Activates SIRT1	Antioxidant, anti-inflammatory; efficacy SIRT1-dependent	[224]
Pterostilbene	HDAC3	Inhibits HDAC3, modulates HDAC3/Nrf1 axis	Reduces neuroinflammation, enhances recovery	[109]
